# Skin ulceration as a complication from unexpected extravasation injury: A case report

**DOI:** 10.1016/j.amsu.2021.102267

**Published:** 2021-03-31

**Authors:** Hock Chin Chong, Kean Khang Fong, Firdaus Hayati

**Affiliations:** a Department of Surgery, Ampang Hospital, Ampang Jaya, Selangor, Malaysia; b Intensive Care Unit, Queen Elizabeth Hospital, Kota Kinabalu, Sabah, Malaysia; c Department of Surgery, Faculty of Medicine and Health Sciences, Universiti Malaysia Sabah, Kota Kinabalu, Sabah, Malaysia

**Keywords:** Extravasation, Iatrogenic disease, Thrombophlebitis, Necrosis

## Abstract

**Background:**

Extravasation injury (EVI) is common, yet it is always underestimated and underreported. Severity varies ranging from thrombophlebitis up to disability. Unrecognised EVI is a potential medicolegal case in medicine.

**Case presentation:**

We experience a 47-year-old lady who developed an unrecognised EVI after being admitted for sepsis. The EVI turned out to be a huge and sloughy skin ulcer. A series of wound debridement with vacuum dressing were conducted until the wound was able to be closed.

**Discussion:**

The EVI can be categorised according to Amjad EVI grading and Loth and Eversmann's EVI classification. Adult EVI tends to be overlooked, especially during critical care because patients cannot complain upon sedation and ventilation. In order to prevent EVI, firstly prevention is better than cure. Secondly, if EVI is recognised early, infusion should be stopped immediately. Thirdly, analgesia is mandatory. Finally, the plastic team needs to be engaged if it is deemed required.

**Conclusion:**

Prevention and early intervention before the occurrence of progressive tissue damage is the key to treatment. Early radical wound debridement and immediate or delayed wound coverage with skin graft or skin flap are indicated in full thickness skin necrosis, persistent pain, and chronic ulcer.

## Highlights

• Unrecognised extravasation injury (EVI) is a potential medicolegal and lawsuit cases in medicine.
• EVI can lead to morbidities such as contracture, nerve damage, joint stiffness, and tendon damage.
• Awareness, prevention and early intervention before the progression of tissue damage is the key of treatment.

## 1 Introduction

Extravasation injury (EVI) is caused by the escape of solution from a vessel into the surrounding structure during intravenous infusion [1]. It is common with reported cases between 0.1 and 6% in adult intravenous therapy [2,3]. Unrecognised EVI can be a medicolegal and lawsuit case in medicine [4]. The extent of tissue injury depends on extravasation volume, site of cannula, patient factor, and agent osmolarity and toxicity [5,6]. Most common places for complicated EVI to occur include the forearm, antecubital fossa, dorsum of the hand, and foot due to the lack of substantial fat tissue barrier. Not only can it lead to a full thickness skin loss, but it can also cause severe damage to the underlying structure [2]. We highlight a 47-year-old lady with an unexpected EVI and discuss the management of complicated EVI in adults in order to prevent disability and dysfunction. This work has been reported in line with the SCARE criteria [7].

## 2 Case presentation

A 47-year-old lady with underlying diabetes mellitus and hypertension, presented with lethargy and progressive body weakness for 2 weeks, shortness of breath, and orthopnoea for 3 days. She was admitted in the Intensive Care Unit (ICU) and treated as Gullien-Barriere Syndrome with AMAN variant and Streptococcus Hemolyticus bacteraemia after a couple of investigations. Intravenous immunoglobulin, as well as 2 courses of antibiotics (ceftazidime and amoxicillin clavulanate, followed by meropenem), was administered through a central venous catheter. In the second week of ICU admission, she developed an upper gastrointestinal bleeding and required inotropic support and endoscopy. She developed a rash over the upper trunk after intravenous sedations (benzodiazepine, propofol and fentanyl), so blood transfusion was given. Subsequently, the allergic reaction resolved after chlorpheniramine was given.

**Fig. 1:**
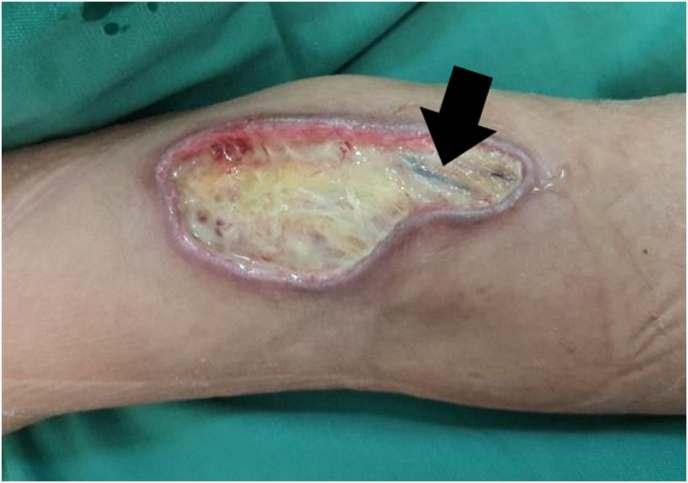
Wound debridement done after 3 weeks dressing of the right cubital fossa blister wound; wound was sloughy and the basilic vein was exposed (arrow).

**Fig. 2:**
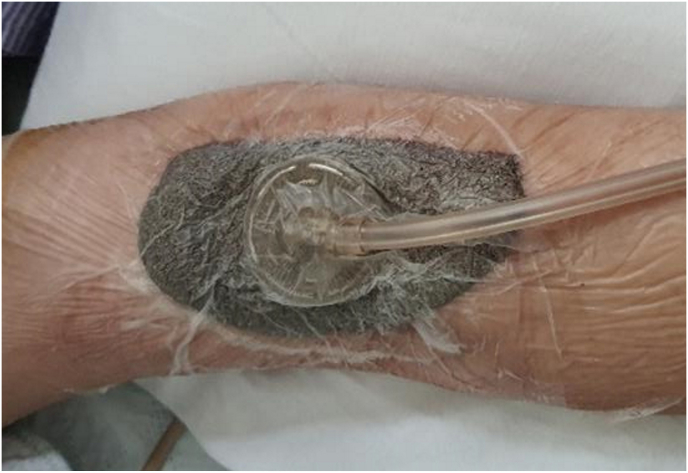
Vacuum dressing of the right cubital fossa; wound after basilic vein ligation; and repeat wound debridement as non-healing wound with regular dressing.

**Fig. 3:**
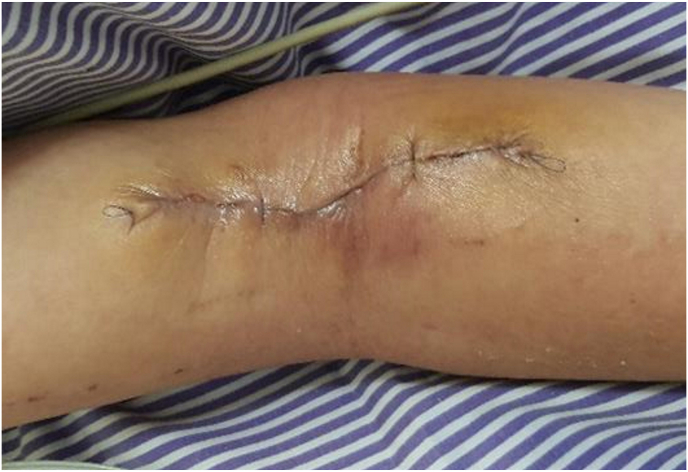
Wound debridement; delayed wound closure was done once the wound is clean.

Two days later, we noticed a 6 × 5 cm non-tender bluish blister over the right cubital fossa at the previously placed intravenous cannula. The wound was dressed using purilon gel and foam every alternate day. After 3 weeks, the wound became sloughy and bedside wound debridement was done (Fig. 1). The wound measured about 8 × 5 cm with exposed subcutaneous fat. It did not heal despite regular dressing; thus, the patient was referred to the plastic team for further wound management. Wound debridement, basilic vein ligation, and vacuum dressing were done by the plastic team in the operation theatre (Fig. 2). After 1 cycle of vacuum dressing and no organism growth from the tissue culture, another wound debridement was performed with a delayed wound closure (Fig. 3). Postoperative recovery was complicated with congested and swollen tissue, which was treated conservatively by upper limb elevation. The oedema subsided eventually and sutures were removed at day-14 post operation. The patient was able to move her right upper limb in full range of movement and later transferred out of the ICU to the neuromedical ward. Later she was discharge and followed up in plastic clinic in 2 weeks, then 1 month and 3 months. Her wound was healed well and there is no functional disability.

## 3 Discussion

Patients with EVI initially present with symptoms similar to thrombophlebitis, such as oedema, pain, and erythema [6]. If symptoms persist for more than 24 hours, it is considered a severe EVI, which can potentially develop ulceration or even necrosis, which may appear as late as weeks later [6]. The worst complications of EVI can result in disability, namely contracture, nerve damage, joint stiffness, and tendon damage [3]. Therefore, further damage can be prevented if EVI is detected earlier [5]. On the other hand, in complicated EVI, early aggressive wound debridement is suggested because chronicity of the wound will increase morbidity and cause more functional damage [3].

Most of the literature focused more on chemotherapy drugs and paediatric EVI, but occurrences in adults tend to be overlooked. The severity of many EVI cases may have been underestimated; therefore, they have been underreported [1,5]. EVI is more difficult to be recognised in ventilated and sedated patients, as patients would be unable to complain about the condition to healthcare workers [3]. In our case, the severity of EVI was underestimated as it initially exhibited partial thickness, before eventually turning into full thickness skin loss. The cause of EVI could not be identified as the cannula was removed earlier and the patient was on the central venous line during the discovery of EVI. The potential medications that may have caused the EVI in this case were propofol, dextrose, and packed cells.

There are 2 types of grading and classification for EVI according to Amjad EVI grading and Loth and Eversmann's EVI classification, respectively [4,6]. These two guidelines would help us to identify and classify the EVI severity, allowing for the determination of which treatment to be administered. Our patient had a grade 4 severe EVI. However, the extravasation volume could not be determined and the patient could not complain about any pain since she was intubated. Generally, the management of EVI can be categorised into (1) prevention; (2) non-pharmacological treatment; (3) pharmacological treatment; and (4) surgery [1,3,4,6].

First, prevention is better than cure. Prevention should always be practiced by healthcare workers during cannulation and drug administration, especially with regard to vesicant or irritant drugs. For cannulation, a good vein should be chosen; cannulation should start from the distal to proximal order in the same vein, while joint sites and lower limbs should be avoided. Next, before drug administration, the patency of cannula should be checked, the intraluminal pressure in the infusion pump should be monitored, and the drug should be diluted in a compatible solution. If the drug is vesicant or irritant, it should be given in a slow rate or a large vein, before any other drug can be given. After all, a regular daily monitoring is vital in detecting early EVI to prevent progressive tissue damage.

Second, if EVI is recognised early, infusion should be stopped immediately. Extravasated drug should be aspirated, including 3–5ml blood followed by reversal agents/antidote according to the extravasated drug. If the extravasated drug is indeterminate, 0.9% normal saline or hyaluronidase subcutaneous injection can be given to dilute and disperse the drug into surrounding tissues, followed by egression of irrigation fluid through several stab incisions in the surrounding skin. Squeeze technique can be used in a distal to proximal direction to squeeze out the extravasated drug in case the catheter had been removed. Many other measures can be done, such as elevation the EVI limb to reduce the swelling and promote the lymphatic reabsorption of extravasated drug; warm compression for vasoconstrict agent or specific drug (phenytoin, vinca alkaloids, and contrast media); cold compression for vesicant or irritant drugs for 15–20 minutes, 3–4 times per day for the first 48–72 hours after EVI occurrence; area marking or serial digital photograph for monitoring; and regular dressing. Conservative management is preferred until the lesion has evolved for at least 1 week.

Third, analgesia is important because EVI can be as painful as a burn wound. In an infected wound, antibiotics should be used to treat the infection. However, prophylactic antibiotic is not recommended. Finally, liposuction with/without saline flush out should be done within 24 hours after high volume EVI. Plastic team referral for early radical wound debridement and immediate or delayed wound coverage with skin graft or skin flap should be done if full thickness skin necrosis, persistent pain, and chronic ulcer occurred. Fasciotomy is indicated in EVI patient with compartment syndrome.

Adult extravasation injury tends to be overlooked, especially during critical care because patients cannot complain upon ventilation, physicians focus on more serious morbidity that patients undergo, and dressing covers up the wound. Therefore, healthcare workers should be more vigilant, and cannulation sites should be regularly monitored, even on previous cannulation sites. The prevention measures, as mentioned in table above, should be reinforced in all patient care.

In conclusion, EVI can lead to significant morbidity and disability in the long term. It may easily be underestimated and underreported, especially during critical care. Awareness, prevention, and regular monitoring are vital for cannulation care. In severe EVI, the wound should be monitored regularly. Early radical wound debridement and immediate or delayed wound coverage with skin graft or skin flap are indicated in full thickness skin necrosis, persistent pain, and chronic ulcer.

## Ethical approval

Not related as this is a case report.

## Source of funding

Not available.

## Authors’ contributions

CHC - manuscript preparation, data collection.

KKF - involvement in managing the patient, data collection.

FH - literature search, final review.

## Registration of research studies

This is a case report. No human participants were involved.

## Guarantor

FH.

## Consent

Consent was obtained from the patient.

Provenance and peer review.

Not commissioned, externally peer-reviewed.

## Declaration of competing interest

The authors declare that no relevant or material financial interests exist.

## Appendix

EVI: extravasation injury.

ICU: intensive care unit.
